# Microstructure Control and Performance Evolution of Aluminum Alloy 7075 by Nano-Treating

**DOI:** 10.1038/s41598-019-47182-9

**Published:** 2019-07-23

**Authors:** Min Zuo, Maximilian Sokoluk, Chezheng Cao, Jie Yuan, Shiqi Zheng, Xiaochun Li

**Affiliations:** 1 School of Materials Science and Engineering, University of Jinan, Jinan, 250022 People’s Republic of China; 2 Department of Mechanical and Aerospace Engineering, University of California Los Angeles, California, 90094 United States; 3 Department of Materials Science and Engineering, University of California Los Angeles, California, 90094 United States

**Keywords:** Other nanotechnology, Metals and alloys

## Abstract

Nano-treating is a novel concept wherein a low percentage of nanoparticles is used for microstructural control and property tuning in metals and alloys. The nano-treating of AA7075 was investigated to control its microstructure and improve its structural stability for high performance. After treatment with TiC nanoparticles, the grains were significantly refined from coarse dendrites of hundreds of micrometers to fine equiaxial ones smaller than 20 μm. After T6 heat treatment, the grains, with an average size of 18.5 μm, remained almost unchanged, demonstrating an excellent thermal stability. It was found that besides of growth restriction factor by pinning behavior on grain boundries, TiC nanoparticles served as both an effective nucleation agent for primary grains and an effective secondary phase modifier in AA7075. Furthermore, the mechanical properties of nano-treated AA7075 were improved over those of the pure alloy. Thus, nano-treating provides a new method to enhance the performance of aluminum alloys for numerous applications.

## Introduction

Recently, 7000-series Al alloys, especially Al alloy 7075 (AA7075), have drawn considerable attention due to their exceptionally high strength-to-weight ratios in structural components for the aerospace and automotive industries^1,2^. However, some long-standing problems still exist, hindering their widespread applications. For example, the mechanical properties of these alloys experience a noticeable reduction at elevated temperatures due to grain and precipitate coarsening^3,4^. Hot cracking often occurs during the solidification of 7000-series Al alloys due to their wide solidification ranges^5^. Moreover, a significant number of intermetallic compounds and eutectic phases composed of Zn, Mg, Cu, and Al usually distribute along grain boundaries, resulting in a remarkable reduction in mechanical properties after heat treatment and hot deformation processes^6,7^. Thus, it is important to address these problems to improve the performance and extend the application space for these alloys.

Many studies have been conducted to control the microstructure of AA7075 in order to optimize its performance for wider applications. Various techniques have been attempted, such as severe plastic deformation^8–10^, grain refinement^11,12^, and so on. With the addition of boron, the grains in Al-Zn-Mg-Cu alloys produced by strain-induced melt activation (SIMA) were significantly refined but still coarser than 60 μm^13^. Ebrahimi *et al*.^14^ reported that the addition of Ti could refine the grain size of the as-cast Zn-rich Al-Zn-Mg-Cu alloy from 859 μm to 46 μm, and Al_3_Ti particles could effectively pin dislocations and grain boundaries during solution treatment. The combination of Sc and Zr in Al-Zn-Mg-Cu alloys is an effective route towards improving their recrystallization resistance (and thus thermal stability), attributed to the formation of the intermetallic compound Al_3_(Zr, Sc)^15^. Rogal *et al*.^16^ found that with Sc and Zr additions, the grains in AA7075 could be refined to less than 30 μm. After T6_1_ or T6_2_ heat treatment, the grains in AA7075ScZr grew coarser than those in the as-cast alloy. Wen *et al*.^17^ also reported that, with the addition of Zr, the grain size in the Al-Zn-Mg-Cu alloy still grew after a prolonged homogenization process.

Recently, nanotechnological methods, such as using a low percentage of nanoparticles to modify alloys (i.e. nano-treating), have gained increasing attention in metallurgy due to their revolutionary capabilities, such as microstructural control and property tuning^18–23^. The resultant properties of the nano-treated alloys would be highly dependent on the type, size, and distribution of the modified precipitate particles^24^.

TiC nanoparticles (NPs) are attractive reinforcements for Al alloys due to their high melting point, high stiffness, high hardness, good thermal stability, and low thermal expansion coefficient. Furthermore, TiC is an excellent heterogeneous nucleation agent for Al due to a similar face centered cubic (FCC) crystal structure and small lattice mismatch with Al^25^. In this paper, a nano-treatment approach with a low percentage of TiC nanoparticles was applied to process and modify AA7075. It also reports the effect of this approach on the solidification rate and the control mechanism of the microstructure in nano-treated AA7075. The novel nano-treatment method would open significant new opportunities for microstructural control and property enhancement of many, if not all, metals and alloys.

## Results and Discussion

### Microstructures of as-cast AA7075 before and after nano-treating

**Figure 1:**
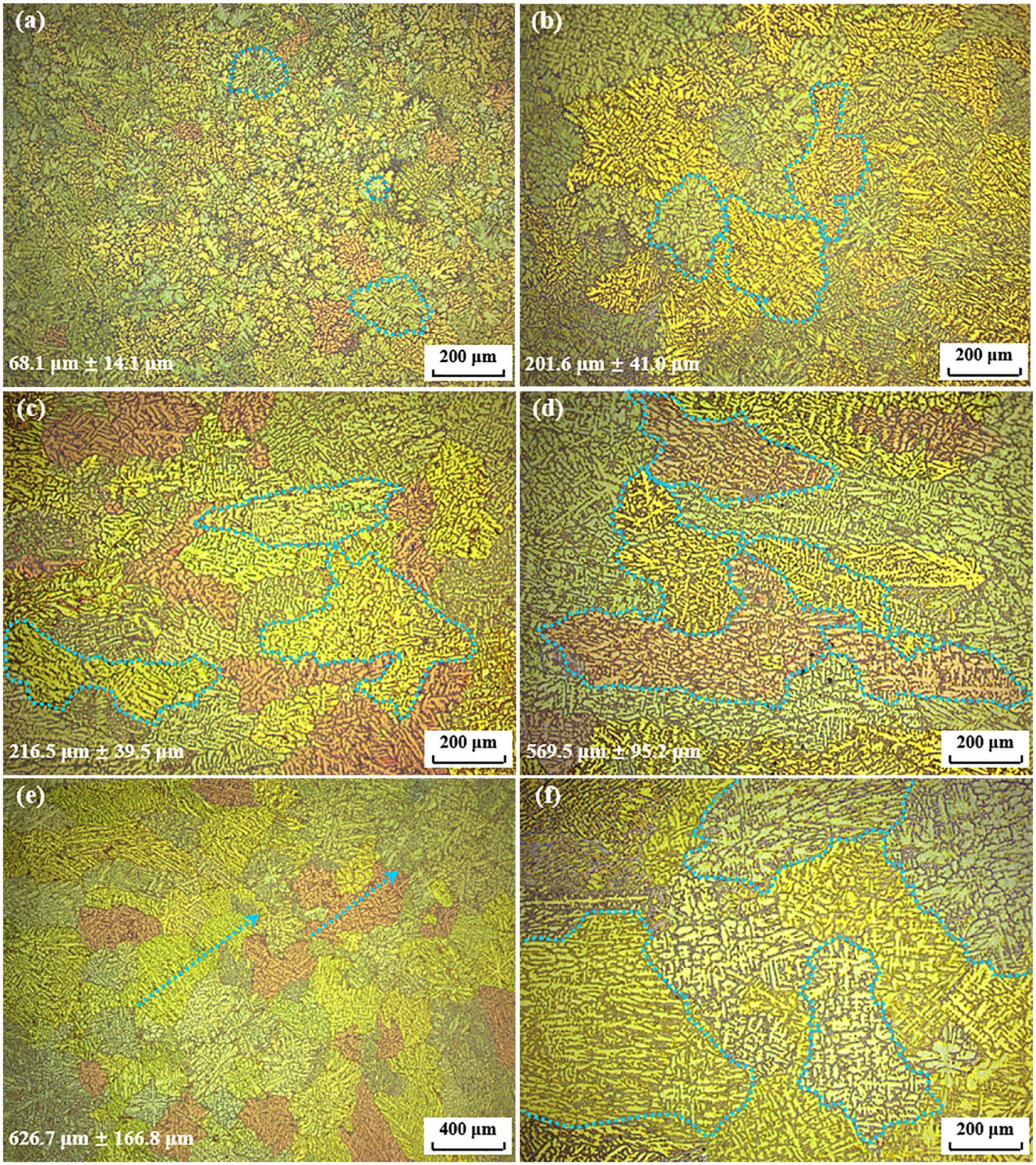
Typical microstructures of pure AA7075 alloys with different cooling rates. (**a**) 197.5 K/s; (**b**) 87.8 K/s; (**c**) 29.5 K/s; (**d**) 20.3 K/s; (**e**,**f**) 14.8 K/s.

Figure 1 shows the typical microstructures of pure AA7075 alloys solidified in the copper wedge mold. It is well known that the cooling rate has a significant influence on the microstructural characteristics of metallic alloys, which in turn affect their mechanical properties. As indicated by the arrows in Fig. 1, the microstructures of these alloys are composed of dendrites that are sensitive to the cooling rate. When the cooling rate decreases from 197.5 K/s to 14.8 K/s, the grains become rather coarse, growing from 68.1 μm ± 14.1 μm to 626.7 μm ± 166.8 μm in length. In the sample cooled at 14.8 K/s, plenty of evenly developed dendrites were observed, as shown in Fig. 1(e).

**Figure 2:**
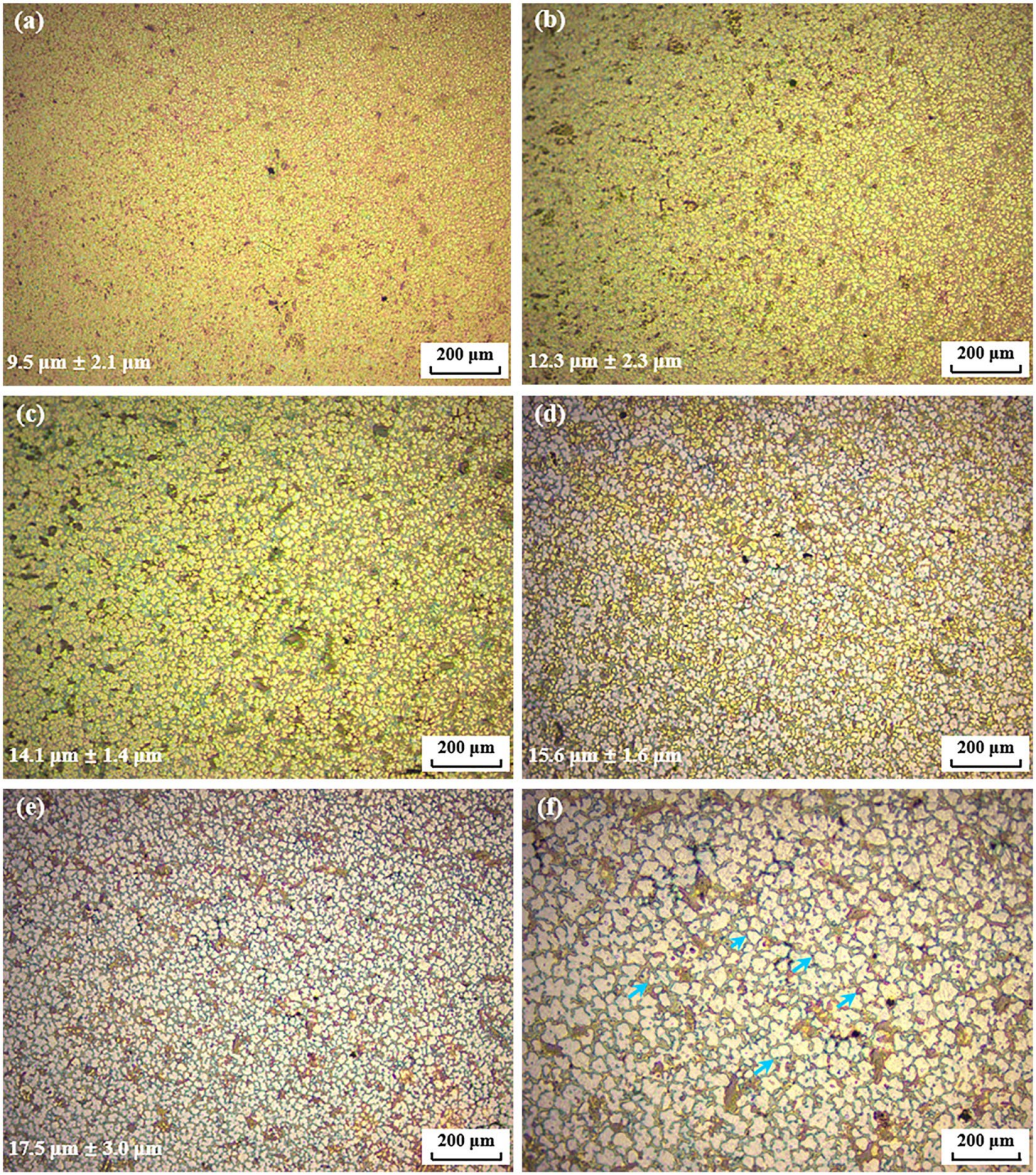
Typical microstructures of nano-treated AA7075 with TiC NPs under varied cooling rates. (**a**) 197.5 K/s; (**b**) 87.8 K/s; (**c**) 29.5 K/s; (**d**) 20.3 K/s; (**e**,**f**) 14.8 K/s.

In order to investigate the nano-treating effect of TiC on AA7075, a sample containing 1 vol.% of TiC nanoparticles was fabricated. The typical microstructure of this sample is illustrated in Fig. 2. By statistical analysis, it was determined that the microstructure of these nano-treated alloys was composed of fine equiaxed grains with a mean size of less than 20 μm. For the sample with a solidification rate of 14.8 K/s, the average size of Al grains was only 17.5 μm ± 3.0 μm, as clearly indicated in Fig. 2(e). In comparison, the coarse dendrites with the same cooling rate in the pure alloy measured up to 626.7 μm. Based on this observation, it can be concluded that the grain size of AA7075 can be effectively refined by TiC nanoparticles.

### Microstructures of heat-treated AA7075 before and after nano-treating

**Figure 3:**
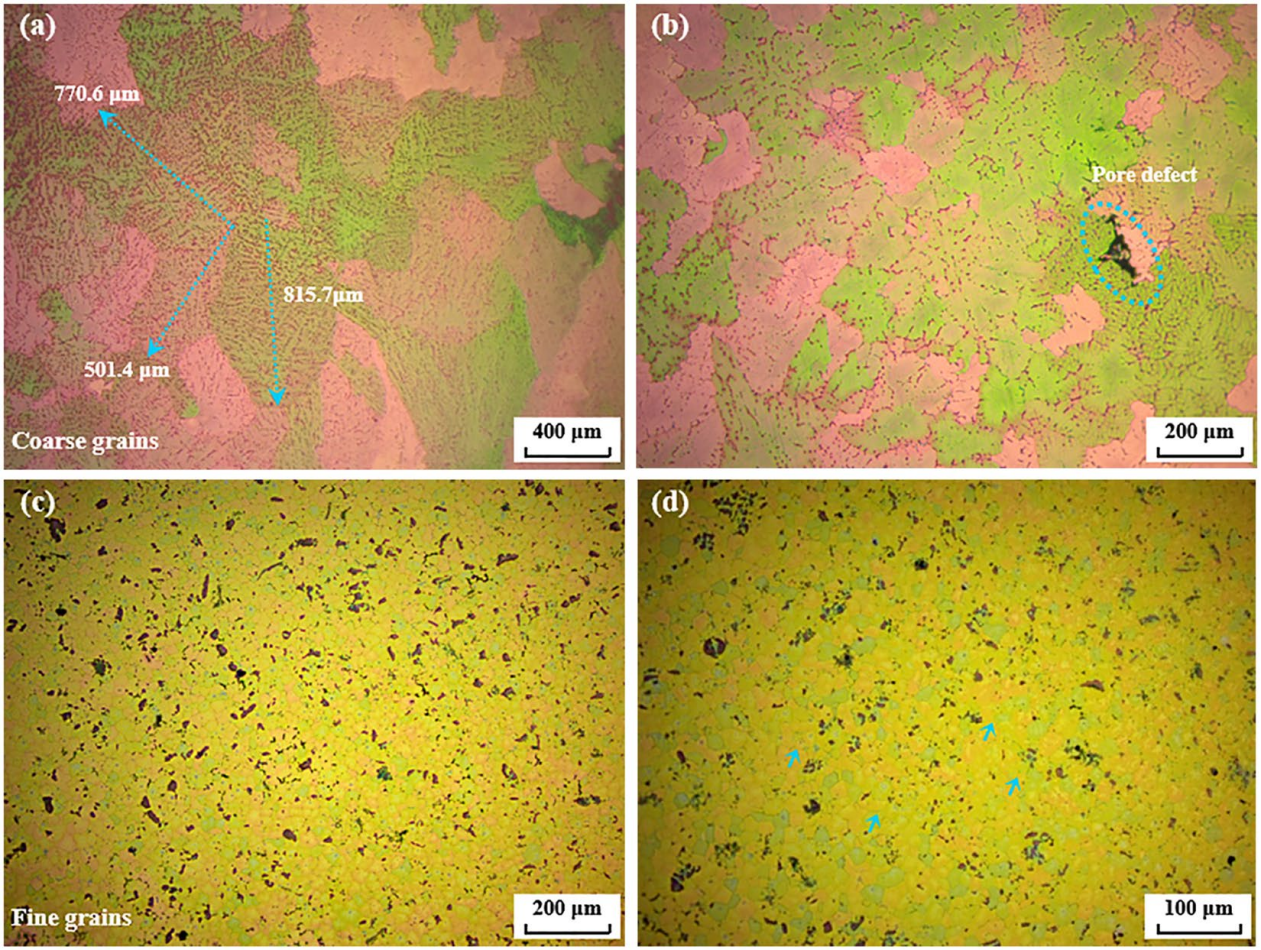
Typical microstructures of AA7075 (sample with cooling rate as 14.8 K/s) after T6 heat treatment. (**a**,**b**) Pure sample; (**c**,**d**) nano-treated sample.

**Figure 4:**
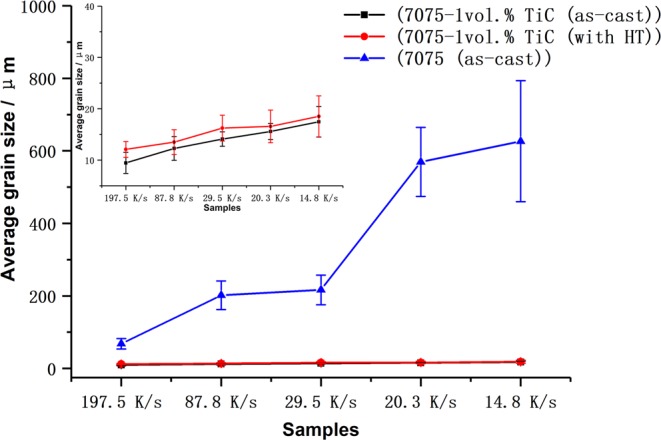
The variation in average grain sizes of AA7075 alloys before and after nano-treating. The embedded image is the partial enlargement of mean grain sizes of nano-treated AA7075, indicating a superior thermal stability of grain size before and after heat treatment. However, the grains in basic AA7075 alloys grow even coarser after heat treatment as clearly illustrated in Fig. 3(a,b).

It is well known that a fine grain structure can enhance the mechanical properties of bulk Al alloys^8^. As a typical precipitation-hardening Al alloy, the microstructural characteristics of heat-treated AA7075 alloys with and without nano-treating are shown in Fig. 3, which is the sample of bulk cast AA7075 with a solidification rate of 14.8 K/s. It can be found that after heat treatment the difference of grain sizes between basic AA7075 and nano-treated AA7075 alloys become even greater. As clearly indicated by arrows in Fig. 3(a), the dendrites in the pure alloy grow much coarser with sizes of up to hundreds of micrometers. In comparison, the microstructure of heat-treated AA7075 fabricated by nano-treatment shows an average grain size of 18.5 ± 4.0 µm, which is quite similar to that of the as-cast nano-treated sample, indicating a superior thermal stability of grain size with nano-treatment. The detailed average grain sizes and standard deviations in AA7075 alloys (according to different cooling rates) are illustrated in Fig. 4. Based on this thermal stability study, it can be speculated that the nano-treatment provides new opportunities for high performance and thermally stable Al alloys.

### Mechanical properties of AA7075 before and after nano-treating

**Figure 5:**
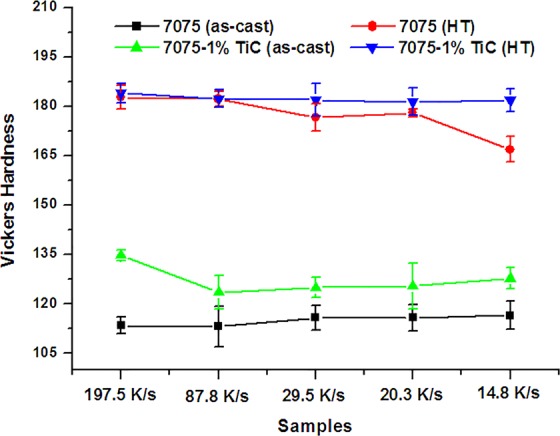
Variations in Vickers Hardness of AA7075 alloys with different cooling rates.

With the excellent microstructural refinement through nano-treatment, the mechanical properties of AA7075 were also improved. The tensile data of series of AA7075 alloys can be found as Supplementary Fig. S1. As shown in Fig. 5, the Vickers hardness of the as-cast nano-treated samples were enhanced from 110 HV to about 130 HV, and the samples with finer grains have relatively higher hardness. After heat treatment, the hardness of nano-treated AA7075 alloys increased to about 180 HV. However, the hardness of the pure alloy seems to be more sensitive to the grain size, which has been directly influenced by the cooling rate, as shown in Fig. 4. When the grains grew coarser with the decrease in cooling rate, the hardness of these samples gradually decreased from over 180 HV to about 165 HV. In contrast, the hardness of the nano-treated samples after heat treatment increased to about 188 HV and remained stable despite different cooling rates, which suggests that the nano-treated samples were thermally stable and insensitive to cooling rate.

Ramakoteswara Rao *et al*.^26^ synthesized 7075 composite alloys reinforced with 2 to 10 wt.% of TiC particles through stir casting. They reported that the best Vickers hardness obtained was 115.9 HV for as-cast AA7075 with 8 wt.% of TiC particles. Wu *et al*.^27^ studied the influence of *in-situ* TiC particles’ form, distribution state, and content on the microstructure and mechanical properties of TiC-AA7075 composites, and they found that the highest hardness achieved was 108 HB by composite alloys containing 8 wt.% TiC. It’s worth noting that through highly efficient nano-treating, AA7075 with better mechanical properties can be obtained with a rather low content of TiC NPs.

### Mechanism behind the nano-treatment of AA7075

**Figure 6:**
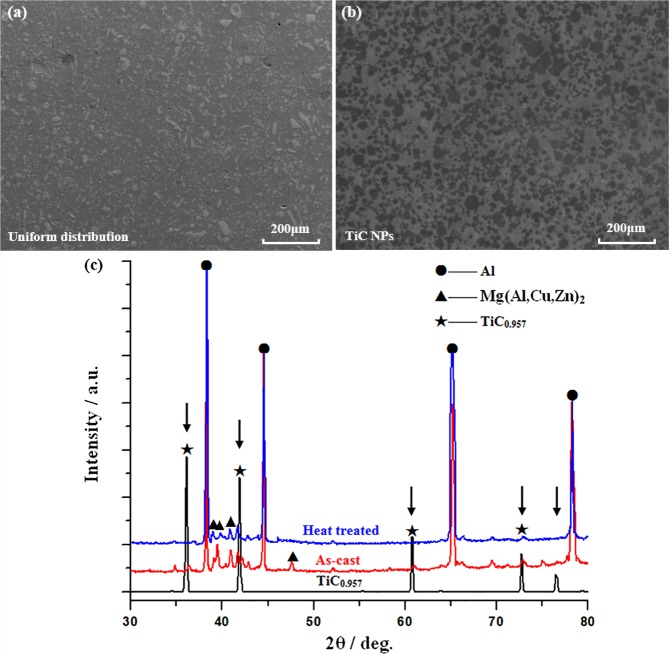
(**a**,**b**) FESEM characterizations of Al–TiC master alloy prepared by a molten salt-assisted processing method. (**c**) XRD patterns of AA7075 alloys before and after nano-treating with TiC NPs.

Figure 6(a,b) shows typical FESEM of Al-TiC master nanocomposite prepared by a molten salt-assisted processing method. It can be found that TiC NPs were well introduced into aluminum matrix and mainly existed in the form of micrometer scale clusters. From partial enlargement, it can be found TiC NPs were rather well dispersed in nanoparticles rich area, which would have a further improvement for the nano-treatment effect of metallic alloys. Figure 6(c) shows the XRD patterns of nano-treated AA7075 alloys before and after heat treatment and the corresponding standard diffraction peaks of TiC_x_ (x = 0.957). Due to the carbon vacancies^28,29^, the C/TiC atom ratio in TiC was not 1:1. Instead, it usually fell in the wide x range from 0.49 to 0.98 without a change in crystal structure. According to the XRD patterns illustrated in Fig. 6, it was observed that AA7075 treated with TiC NPs is mainly composed of three phases, including Al, Mg(Al,Cu,Zn)_2_, and TiC. The diffraction lines of nano-treated AA7075 alloys before and after heat treatment both exhibit peaks at 36.41°, 41.68°, 61.21°, and 72.85°, corresponding to the (111), (200), (220), and (311) planes of the FCC TiC respectively; This could provide evidence for the existence of TiC particles in AA7075. Furthermore, the diffraction peaks of TiC can also be detected in the heat-treated sample, meaning that TiC can stably exist in this system at elevated temperatures.

**Figure 7:**
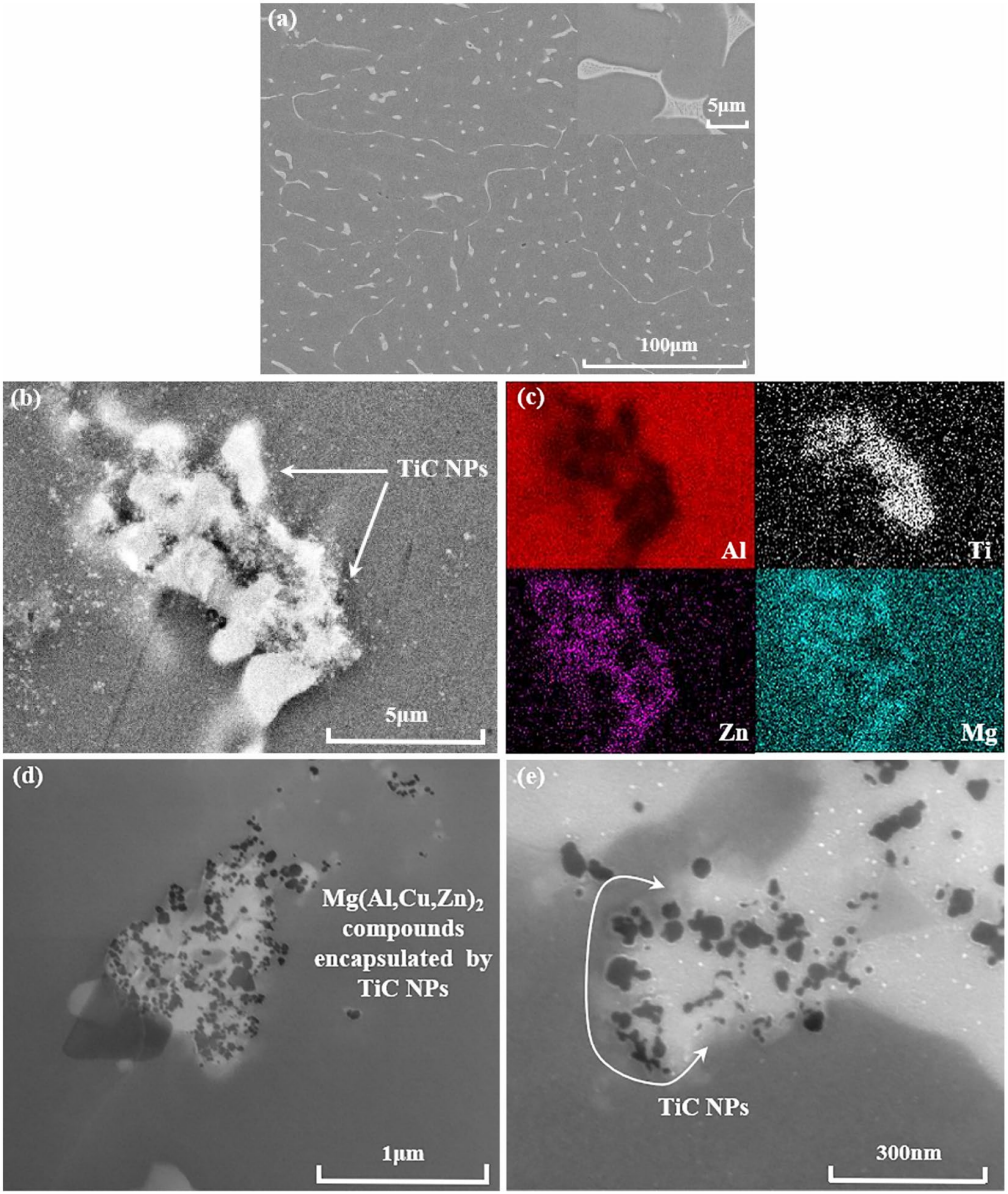
FESEM characterizations of secondary Mg(Al,Cu,Zn)_2_ phases in AA7075 alloys before and after nano-treatment. (**a**) Secondary phase in pure AA7075 alloys (partial enlargement illustrated in the upper right corner); (**b**) modified secondary phase in AA7075 by TiC NPs; (**c**) the X-ray images for elements Al, Ti, Zn and Mg in (**b**); (**d**,**e**) modification features of secondary phases by TiC NPs.

In order to investigate the influence of nano-treatment on Mg(Al,Cu,Zn)_2_ in detail, FESEM characterization of secondary phases in AA7075 alloys are studied. As clearly illustrated in Fig. 7(a), it is observed that the coarse secondary phases tend to precipitate along grain boundaries, especially in the triple junction of them, which would cause solidification cracks and lead to the failure of metallic alloys. With the presence of TiC NPs, the secondary phase is segmented, resulting in finer and shorter features with uniform distribution. In combination with this encapsulation attachment structure, the low mismatch between $(\overline{1}{11}_{{\rm{TiC}}})/(1\overline{{\rm{2}}}{{\rm{10}}}_{{{\rm{MgZn}}}_{{\rm{2}}}})$ interfaces as 5.6% could further affiliate with secondary phases and effectively modify eutectic compounds^30^. By effective nano-treatment of TiC NPs, equiaxed Al grains and fine divorced eutectic features of AA7075 were obtained, suggesting the significant improvement of mechanical properties.

**Figure 8:**
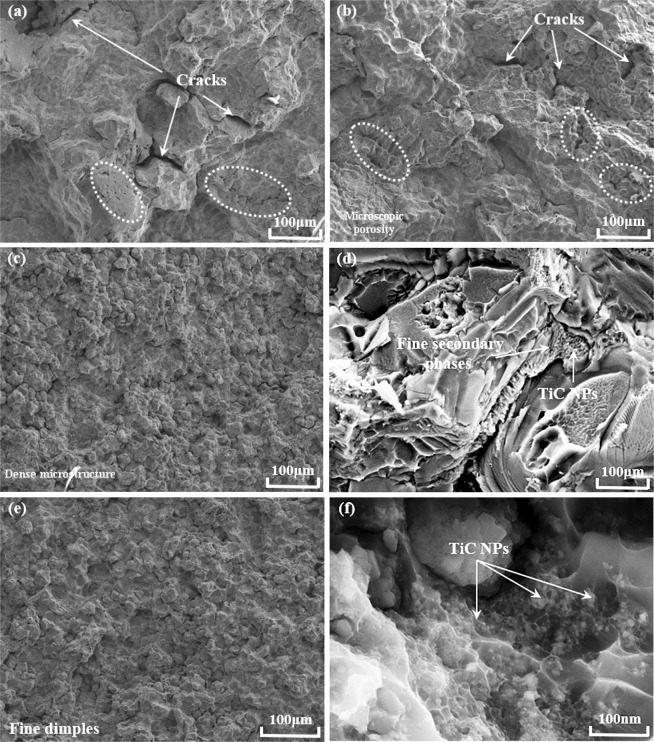
FESEM images of the fracture surfaces of AA7075 alloys with a cooling rate of 14.8 K/s. (**a**,**b**) AA7075 before and after heat treatment. Cracks and microscopic porosities were clearly observed and would lead to the failure of alloys; (**c**,**d**) nano-treated AA7075 (as-cast); (**e**,**f**) nano-treated AA7075 after heat treatment. With nanotreatment by TiC NPs, AA7075 alloys with dense microstructure were obtained.

To obtain more structural characteristics of nano-treated AA7075, the fracture surfaces of AA7075 alloys were analyzed by FESEM and are shown in Fig. 8. For the pure alloy, before and after heat treatment, it can be noticed that the fracture surfaces of both the samples consist of a large quantity of cleavage facets. As indicated in Fig. 8(a,b), the coarse Al grains and Mg(Al,Cu,Zn)_2_ intermetallic compounds that precipitated along the Al grain boundaries can be clearly observed. Furthermore, there are some microscopic porosities detected between coarse dendrites, which might be caused by the hindrance influence of continuous eutectic phases on the flowability during solidification process. After heat treatment, besides many dimples, coarse penetrative cracks appeared, which caused the sample to fail. In comparison, the microstructure of nano-treated AA7075 was much finer and more homogeneous. Many fine dimples were clearly observed in Fig. 8(c–f). Through the enlarged images shown in Fig. 8(d,f), secondary phases precipitated with the existence of uniformly dispersed TiC nanoparticles, significantly modifying their appearance. With the significant refinement of aluminum and modification of the secondary phase, the mechanical properties of nano-treated AA7075 would be obviously improved^31^.

De Cicco and Li *et al*.^32^ raised the nucleation catalysis effect of nanoscale inoculants in aluminum alloys and found that the undercooling would reduce due to the effective nucleation on the nanoparticles’ surfaces. According to different levels of undercooling caused by nanoparticles of various sizes, they reported that nanoparticle encapsulation might occur during the adsorption of the initial crystal layer, which could further promote the refinement effect of nanoparticles on aluminum grains. Meanwhile, the effective grain boundary pinning by nanoparticles due to strong bonding with the matrix has a further promotion in restriction growth of aluminum grains^33^.

**Figure 9:**
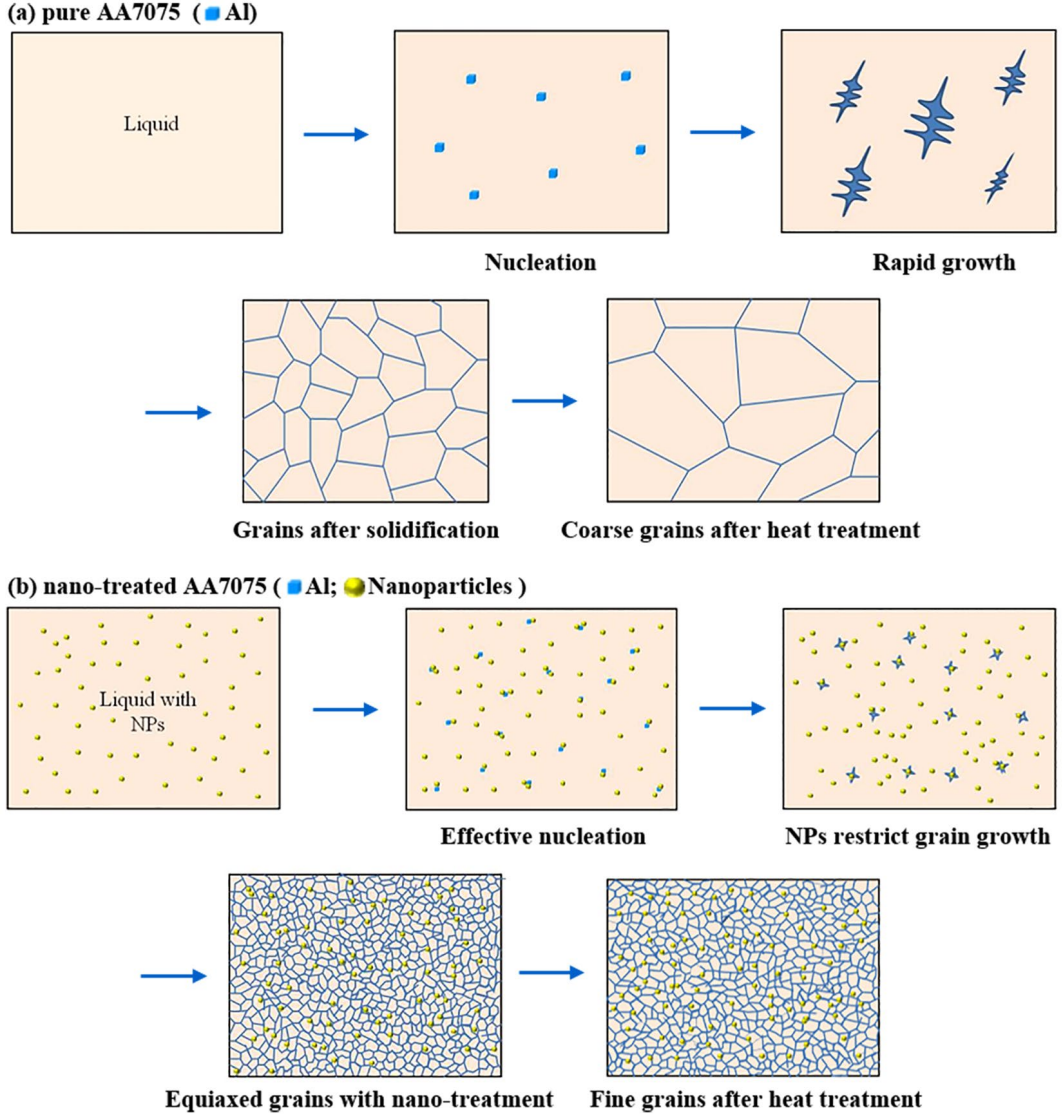
The schematic diagrams of solidification processes of AA7075 alloys. (**a**) Pure alloy; (**b**) nano-treated alloy with TiC NPs.

Therefore, the solidification process of nano-treated AA7075 with TiC NPs can be proposed as follows, and the corresponding schematic diagram is shown in Fig. 9. TiC NPs can act as heterogeneous nucleation sites for primary Al grains due to the small lattice mismatch between Al and TiC^34,35^ as illustrated in Supplementary Fig. S2. According to ref.^36^, the growth restriction factor Q of a free-growth model is found to be a function of refiner addition level, the chemical composition of alloy and solidification rate. Due to the high concentration and rather uniform distribution of TiC NPs in Al-TiC master nanocomposite, the effective quantity of TiC introduced into AA7075 alloys would be supposed to be rather high, which could have an improvement for the growth restriction effect of nanoparticles. With continuous solidification, some TiC NPs and other alloying elements are pushed to the solidification fronts and TiC NPs are quite effective to pin down grain growth by physical barrier (pushing to the grain boundary) to provide considerable growth restriction factor. Then, the encapsulation structure is formed by nanoparticles outside of the eutectic phases due to high surface activity. By means of the restriction effect of encapsulation geometry and specific interface matching between TiC NPs and secondary phases, these eutectic phases of AA7075 alloys are obviously modified as finer divorced features with good dispersion as shown in Fig. 7. Furthermore, it can be observed that there are many TiC NPs embedded into the secondary phases, as clearly illustrated in Fig. 8(d,f). Based on these results, the microstructure of nano-treated AA7075 is significantly improved, with the aluminum dendrites refined to fine equiaxed grains (less than 20 μm) and continuous lamellar eutectic phases to fine divorced features. After heat treatment, the grain sizes of nano-treated AA7075 are similar to those of as-cast samples, which were attributed to the excellent pinning effect of homogeneous TiC NPs. As a result, the mechanical properties of nano-treated AA7075 with dense microstructure are correspondingly improved due to the excellent refinement of its microstructure^37,38^.

## Conclusions

In summary, a novel nano-treatment approach was applied for microstructural control and performance improvement of AA7075. The effect of cooling rate on the nano-treatment of AA7075 was also investigated.

1. When nano-treated with TiC NPs, the coarse dendritic grains in the as-cast samples were refined to small equiaxed grains smaller than 20 µm in size. During T6 heat treatment, the aluminum grains remained almost the same, indicating that nano-treating instilled an exceptional thermal stability to AA7075.
2. In contrast to the pure alloy, the grain size of nano-treated AA7075 was only slightly affected by solidification rates. With a solidification rate at 14.8 K/s, the average grain size of nano-treated AA7075 was about 17.5 μm.
3. The hardness of the nano-treated alloys was enhanced from 110 HV to about 130 HV. The samples with finer grains showed higher hardness. After T6 heat treatment, the hardness of nano-treated AA7075 alloys significantly increased to about 188 HV.
4. It is believed that besides of growth restriction factor by pinning behavior on grain boundries, TiC nanoparticles also serve as effective heterogeneous nucleation sites and excellent modifiers of the secondary phases to optimize the microstructure, resulting in the improvement of mechanical properties of AA7075.

## Experimental Procedures

Commercially pure Al and high-purity TiC nanoparticles (40–60 nm, US Research Nanomaterials, Inc.) were used to fabricate Al-TiC master nanocomposites by a molten salt-assisted processing method^39–41^. Commercially pure Zn, Mg, Cu, and Cr ingots were added into the Al melt to prepare basic AA7075 alloys, whose chemical composition is listed in Table 1 (all compositions are in wt.% unless otherwise stated).

**Table 1:** Chemical composition of AA7075 (wt.%).

Elements	Zn	Mg	Cu	Cr	Fe	Si	Mn	Al
Concentration	5.50	2.50	1.65	0.20	0.11	0.13	0.10	Bal.

Nano-treatment of AA7075 alloys was carried out as follows. Under argon gas protection, about 200 g of base alloy was re-melted in a graphite crucible with an inner diameter of 100 mm and a height of 150 mm by an electrical resistance furnace at 830 °C for 30 min. Subsequently, the Al-6 vol%TiC master nanocomposite was added into the melt, followed by mechanical stirring for 10 min until it melted and obtained uniform dispersion. Finally, the melt was poured into a copper wedge mold. The cooling rate, *R*, corresponding to the thickness, *d*, of the viewing zone in the mold could be calculated by Eq. (1)^42^ where *K* is in Kelvin, *s* is in seconds, and *d* is in mm. From the bottom to the top of the bulk casting samples, the microstructures were studied at five locations, and their corresponding cooling rates were determined to be 197.5 K/s, 87.8 K/s, 29.5 K/s, 20.3 K/s, and 14.8 K/s, respectively.

1 $$R\approx \frac{1000\,Km{m}^{2}}{s}/{(\frac{d}{2})}^{2}$$

The AA7075 samples were heat treated following the T6 procedure, which included solution treating at 460–480 °C for 1 h, followed by water quenching and then artificial aging at 120 °C for 19 h. Metallographic specimens were cut from the midsection of each cast sample and then mechanically ground, polished, and low–angle ion milled by using Precison Ion Polishing System (PIPS) for 1.5 h to expose typical microstructures and embedded nanoparticles. For ion milling process, the accelerating voltage and milling angle were 4 KV and 4°, respectively. For HRTEM analysis, a sample of approximately 50 nm in thickness was obtained from nano-treated AA7075 (as-cast) using Focused Ion Beam (FIB, Zeiss 1540 XB CrossBeam Workstation) and studied with a Titan 80–200 Aberration-corrected S/TEM (FEI). In order to evaluate the grain size of the alloys, samples were etched for 15–25 s using a mixture of 1.0 g NaOH, 4.0 g KMnO_4_ and 100 ml deionized water. The microstructural characterizations of the AA7075 samples were determined with an optical microscope and a field emission scanning electron microscope (FEI Nova 600) equipped with a focused ion beam system. The average grain size of aluminum was obtained from FESEM images using the image analysis software Image J. The Vickers Hardness tests were conducted with a Microhardness Tester (LM800 AT) under 200 gf with a dwell time of 10 s. For different cooling rates, 20 specimens of pure AA7075 and nano-treated AA7075 alloys before and after heat treatments were tested. By statistic analysis of ten points of each specimen, the Vickers hardnesses of series of AA7075 alloys were obtained.

## Supplementary information

Microstructure Control and Performance Evolution of Aluminum Alloy 7075 by Nano-Treating
