# Supplementary material for: Microstructure Control and Performance Evolution of Aluminum Alloy 7075 by Nano-Treating
Source: Sci Rep. 2019 Jul 23;9:10671. doi: 10.1038/s41598-019-47182-9 (PMC6650472; doi:10.1038/s41598-019-47182-9)
Supplement: Supplementary file 1 — Microstructure Control and Performance Evolution of Aluminum Alloy 7075 by Nano-Treating [file 41598_2019_47182_MOESM1_ESM.docx]

**Microstructure Control and Performance Evolution of Aluminum Alloy 7075 by Nano-Treating**

Min Zuo^a, b^﹡, Maximilian Sokoluk^b, c^, Chezheng Cao^b, c^, Jie Yuan^b, c^, Shiqi Zheng^b, c^, Xiaochun Li^b, c^﹡

^a^ School of Materials Science and Engineering, University of Jinan, Jinan 250022, People′s Republic of China

^b^ Department of Mechanical and Aerospace Engineering, University of California Los Angeles, California, 90094, United States

^c^ Department of Materials Science and Engineering, University of California Los Angeles, California, 90094, United States

﹡ Corresponding authors:

Prof. Min Zuo, + 86-531-8276-5317, mse_zuom@ujn.edu.cn

Prof. Xiaochun Li, +1 310 825-2383, xcli@seas.ucla.edu

The tensile strengths of pure AA7075 alloys and nanotreated AA7075 before and after heat treatment were also examined. As shown in Supplementary Fig. S1 online, nanotreated AA7075 alloys demonstrate better mechanical properties comparing with pure AA7075 alloys before and after heat treatment. It is believed that the effective microstructure control by nano-treatment contributed to the enhancement of mechanical properties. With the addition of TiC nanoparticles, the aluminum dendrites were modified and refined to fine equiaxed grains (less than 20 μm). Also, the continuous lamellar eutectic phases were modified to fine dispersed features. With high efficient nano-treatment of TiC NPs, the improvement of mechanical properties of aluminum alloys could be predicted.


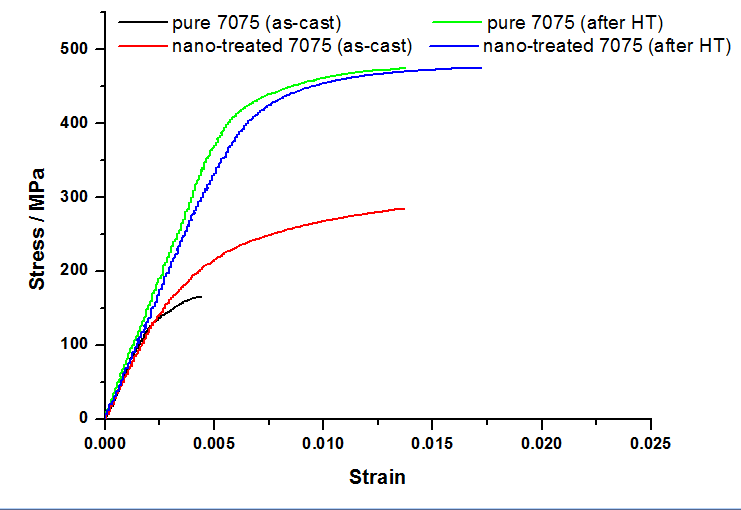


Fig. S1 Engineering stress–strain curve of series of AA7075 alloys solidified in cast iron mold.

To reveal the interface characteristic between Al grains and TiC NPs, high resolution transmission electron microscopic (HRTEM) analysis was examined. Supplementary Fig. S2 shows TEM images of nano-treated AA7075 alloys containing TiC NPs. As clearly illustrated in Fig. S2(a), the clean and strong interface between TiC embedded in MgZn_2_ and Al is obtained and the partial enlarged drawing of the specific interface is shown in Fig. S2(b). By means of selected area electron diffraction (SAED) shown in Fig. S2(c), (111) plane of aluminum grain was indentified. Meanwhile, the observed TiC NPs is oriented towards the zone axis. Furthermore, (111) and (200) planes of TiC nanoparticle are identified, the angular difference between which planes is observed as 54.7°. Based on HTTEM study, it can be found that the good interfacial integrity between aluminum grains and TiC NPs has been obtained.


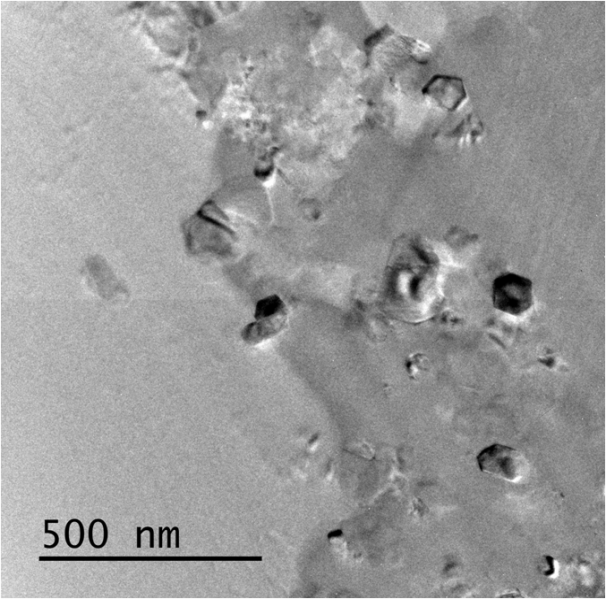

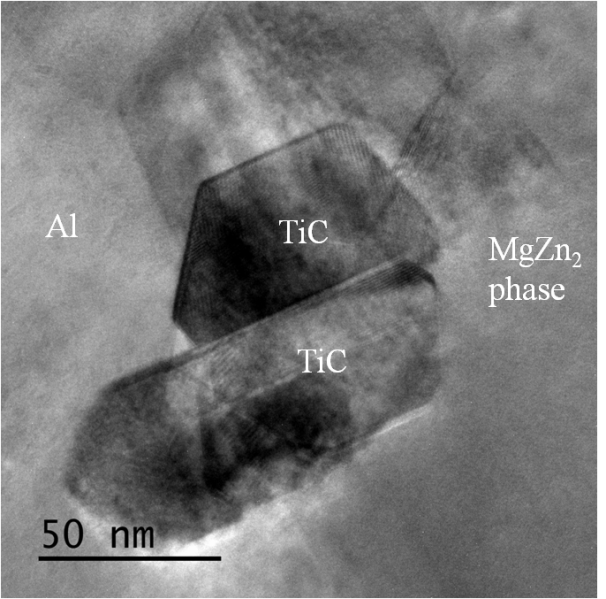


**(b)**

**(a)**


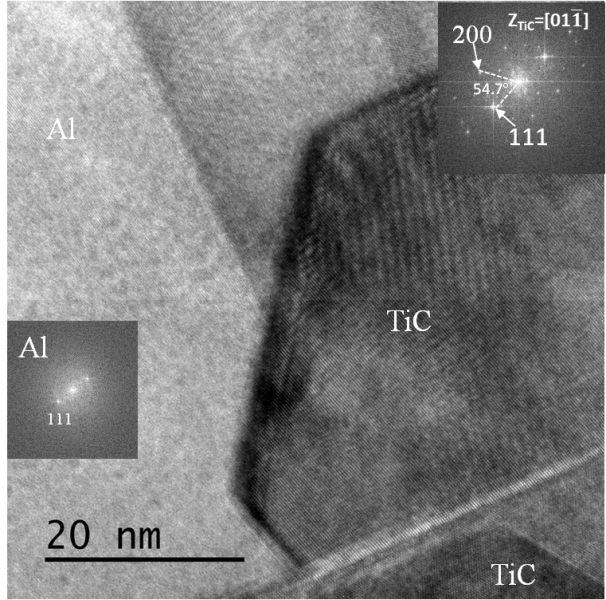


**(c)**

Fig. S2 High resolution TEM images of interface between TiC NPs and aluminum grains in nano-treated AA7075 alloys.
